# An Integrated eDiagnosis Approach (IeDA) versus standard IMCI for assessing and managing childhood illness in Burkina Faso: a stepped-wedge cluster randomised trial

**DOI:** 10.1186/s12913-021-06317-3

**Published:** 2021-04-16

**Authors:** Sophie Sarrassat, James J. Lewis, Arsene S. Some, Serge Somda, Simon Cousens, Karl Blanchet

**Affiliations:** 1 Centre for Maternal Adolescent Reproductive and Child Health (MARCH), London School of Hygiene and Tropical Medicine, Keppel Street, London, WC1E 7HT UK; 2 Y Lab, the Public Services Innovation Lab for Wales, School of Social Sciences, Cardiff University, Cardiff, UK; 3 Centre Muraz, Bobo-Dioulasso, Burkina Faso

**Keywords:** Integrated Management of Childhood Illness, Electronic clinical decision support system, Health care workers’ adherence, Burkina Faso

## Abstract

**Background:**

The Integrated eDiagnosis Approach (IeDA), centred on an electronic Clinical Decision Support System (eCDSS) developed in line with national Integrated Management of Childhood Illness (IMCI) guidelines, was implemented in primary health facilities of two regions of Burkina Faso. An evaluation was performed using a stepped-wedge cluster randomised design with the aim of determining whether the IeDA intervention increased Health Care Workers’ (HCW) adherence to the IMCI guidelines.

**Methods:**

Ten randomly selected facilities per district were visited at each step by two trained nurses: One observed under-five consultations and the second conducted a repeat consultation. The primary outcomes were: overall adherence to clinical assessment tasks; overall correct classification ignoring the severity of the classifications; and overall correct prescription according to HCWs’ classifications. Statistical comparisons between trial arms were performed on cluster/step-level summaries.

**Results:**

On average, 54 and 79% of clinical assessment tasks were observed to be completed by HCWs in the control and intervention districts respectively (cluster-level mean difference = 29.9%; *P*-value = 0.002). The proportion of children for whom the validation nurses and the HCWs recorded the same classifications (ignoring the severity) was 73 and 79% in the control and intervention districts respectively (cluster-level mean difference = 10.1%; *P*-value = 0.004). The proportion of children who received correct prescriptions in accordance with HCWs’ classifications were similar across arms, 78% in the control arm and 77% in the intervention arm (cluster-level mean difference = − 1.1%; *P*-value = 0.788).

**Conclusion:**

The IeDA intervention improved substantially HCWs’ adherence to IMCI’s clinical assessment tasks, leading to some overall increase in correct classifications but to no overall improvement in correct prescriptions. The largest improvements tended to be observed for less common conditions. For more common conditions, HCWs in the control districts performed relatively well, thus limiting the scope to detect an overall impact.

**Trial registration:**

ClinicalTrials.gov NCT02341469; First submitted August 272,014, posted January 19, 2015.

**Supplementary Information:**

The online version contains supplementary material available at 10.1186/s12913-021-06317-3.

## Background

Currently, more than 75 low- and middle-income countries (LMIC) are implementing the Integrated Management of Childhood Illness (IMCI) strategy on a large scale. However, poor adherence of health care workers (HCWs) to guidelines has often been reported [1, 2], likely due to health system limitations, such as lack of training, coordination and supervision, or low availability of essential medicines and equipment [3–6]. In Burkina Faso, the IMCI strategy was introduced in 2003, but an evaluation conducted in 2011 reported a low coverage of training and poor performance in terms of adherence to guidelines [7].

Recent advances in Information and Communication Technologies (ICT) and the advent of electronic Clinical Decision Support System (eCDSS) could potentially transform health care services in LMICs, for instance by helping HCWs to correctly follow relatively complex charts. However, several reviews reveal the lack of evidence for a scalable and sustainable impact on health indicators [8–12]. In particular, the experience with using such technology to improve adherence to the IMCI guidelines is limited [13–17].

From 2014, Terre des hommes foundation (Tdh), in partnership with the Burkinabe Ministry of Health (MoH), implemented, in primary health facilities of two regions of Burkina Faso, the Integrated eDIagnosis Approach (IeDA), a complex intervention centred on an eCDSS developed in line with national IMCI guidelines, with the objective of improving HCWs’ adherence to the IMCI guidelines. Between 2014 and 2017, an evaluation was performed using a stepped-wedge cluster randomised design by an independent team from the London School of Hygiene and Tropical Medicine (LSHTM), United Kingdom, and Centre Muraz, Burkina Faso. The aim of the evaluation was to determine whether the IeDA intervention increased adherence to the IMCI guidelines and improved clinical assessment, classification, prescription, referral and counselling during under-five child consultations in primary health facilities.

## Methods

### Setting

In Burkina Faso, coverage of key effective interventions for preventing child deaths has steadily increased following the adoption of successive public health policies (e.g. free anenatal care, subsidies for child birth and emergency obstetric care, national distribution of insecticide treated nets, Artemisinin-based Combination Therapy (ACT) for treating uncomplicated malaria at facility and community level, expanded program for vaccination). Consequenttly, in 2015, the under-five mortality rate had declined by 56% compared to 1990, from an estimated 202 deaths per 1000 live births in 1990 to 89 deaths per 1000 live births in 2015 [18]. The government is the main health service provider and managed 83% of facilities within the country in 2014 [19]. The country is divided into 13 regions further subdivided into 63 health districts each with one district or regional hospital. In rural areas, primary health facilities, usually run by one or more nurses with the support of health assistants, are the most common point of care and provide a basic package of outpatient services. In 2014, there were 1824 primary health facilities, corresponding to about one facility per 10,000 inhabitants.

**Fig. 1:**
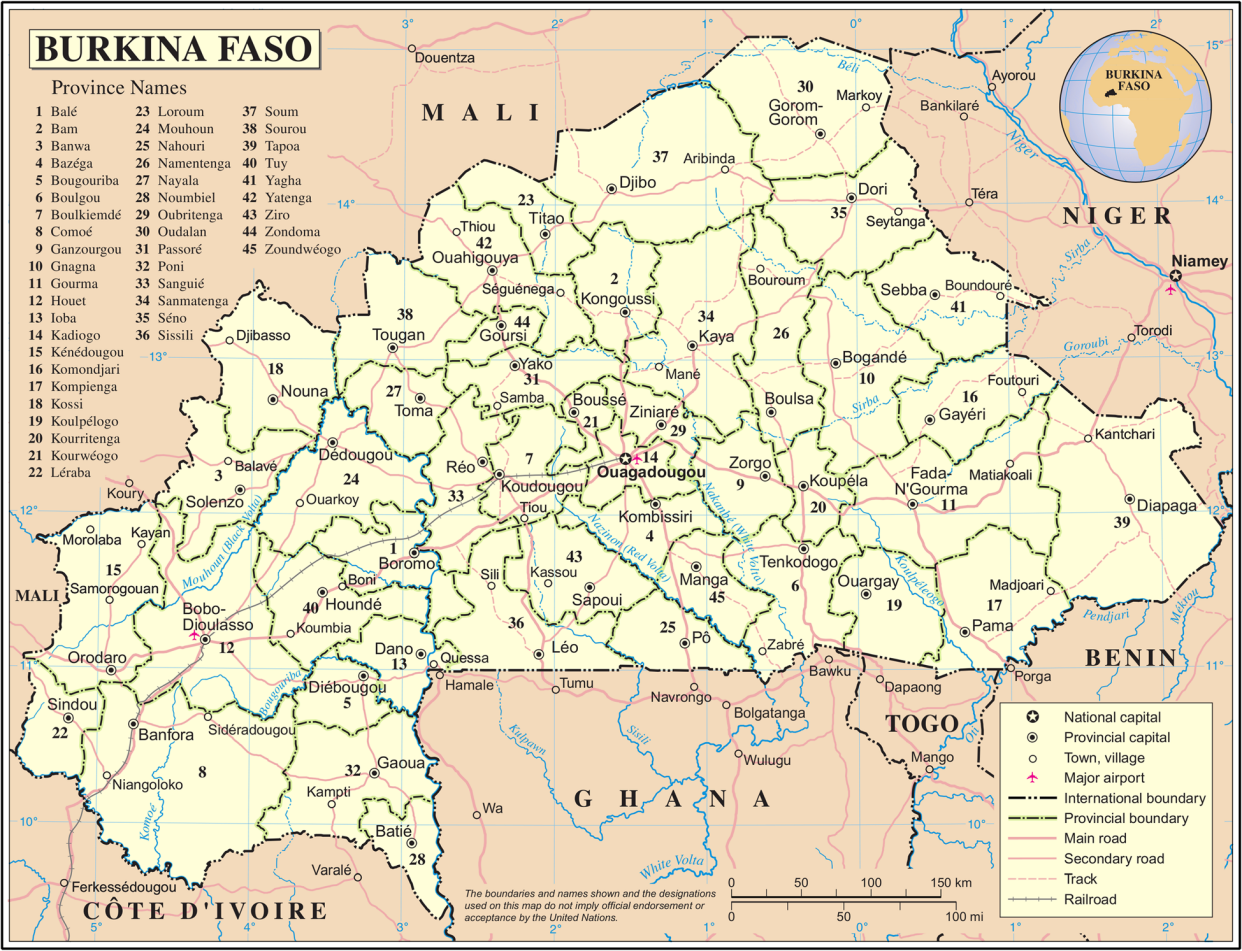
Eight health districts included in the trial. Blue and red circles indicate control and intervention districts respectively. Source: Burkina Faso, Map No. 4230, November 2004, UNITED NATIONS

The evaluation took place in the Boucle du Mouhoun and Nord regions from September 2014 to November 2017. Of the 11 districts in these two regions, three districts were selected by the implementing agencies to pilot the first versions of the eCDSS in 2010 and were therefore excluded from the evaluation, which was restricted to the eight remaining districts (Fig. 1). In addition to IeDA, a performance-based financing (PBF) intervention was independently implemented in four trial districts (Nouna, Solenzo, Gourcy and Ouahigouya districts). From April 2016, free care for under-five children was also introduced by the MoH in all public facilities [20].

### The IeDA intervention

The IeDA intervention comprised five components: 1. An eCDSS provided on tablets to primary health facilities for the management of under-five connsultations: Based on the information recorded by HCWs from the clinical assessment of the child (e.g. body temperature), the eCDSS displays the relevant charts on the screen to guide HCWs through the IMCI national protocol, from the classification (e.g. uncomplicated malaria), through prescription (e.g. first line antimalarial), referral and counselling. During the trial period, several versions were deployed following feedback from users and stakeholders; 2. A six-day training course provided to HCWs on IMCI guidelines and the use of the eCDSS. During the last year of the trial, learning modules with short videos were also available on the eCDSS to support continuous training; 3. A quality assurance coaching system involving team meetings two to four times a year through which health district authorities and HCWs discussed solutions to their local issues (e.g. organisation of care); 4. A supervision system including monthly visits to primary health facilities; 5. A health information system based on data collected through the eCDSS. During the last year of the trial, descriptive dashboards on under-five consultations were developed and shared with the health district authorities and HCWs.

### Evaluation design

Since some components of the intervention could only be delivered at the district level, and rolling out the intervention in a phased manner was more practical for the implementing agencies, the evaluation used a stepped-wedge cluster randomised design, with health districts (“clusters”) receiving the intervention at different time points in a randomised order.

**Fig. 2:**
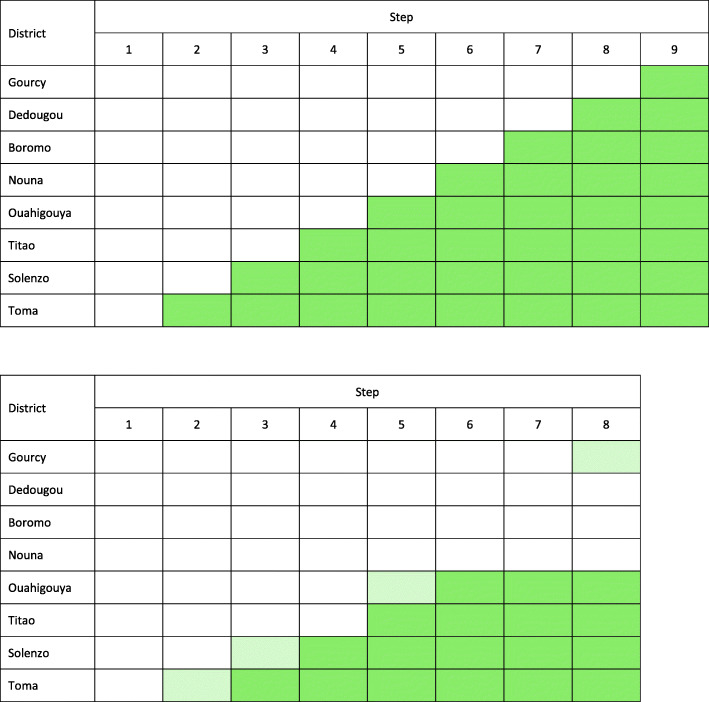
**a** Stepped-wedge design: planned roll-out of the IeDA intervention. **b** Stepped-wedge design: actual roll-out of the IeDA intervention. Districts shaded in dark green had full implementation of the IeDA intervention. Districts shaded in light green had partial implementation of the IeDA intervention (“contaminated” control districts)

Nine steps, one every 4 months, were initially planned, with the first step used as baseline (Fig. 2a). However, funding and logistic issues resulted into delayed roll-out and only four out of eight districts with the intervention implemented. The baseline phase included the first two steps, and during each of the next four steps, from step 3 to step 6, a new district implemented the intervention (Fig. 2b). For the purposes of data collection, ten primary facilities with staff trained in IMCI were randomly selected in each district with stratification on the 2013 annual under-five consultations caseload [21]. Eight rounds of data collection were conducted in total (Fig. 2b).

Full implementation in a district was considered to have been achieved when the eCDSS was provided to all primary facilities and when all HCWs had been trained in its use and IMCI guidelines. In some control districts, data were collected after implementation started but before the full implementation was completed, resulting in some “contamination” of these control districts (Fig. 2b).

### Randomisation and masking

Randomisation was restricted to ensure intervention and control clusters were balanced with respect to region and the PBF intervention. Details of the randomisation procedure used to allocate districts to receive the intervention have been published elsewhere [21]. Randomization was performed by JL, independently of Tdh. The nature of the intervention precluded formal masking of fieldworkers.

The allocation of the intervention to each district was gradually communicated by the research team to the implementing agencies and the list of surveyed facilities was not communicated to reduce the likelihood that more intensive support was provided to those facilities.

### Sample size

The sample size was determined using the method described by Hussey and Hughes [22], assuming a design effect of 2 due to clustering within facilities and a between cluster coefficient of variation of 0.3. With a harmonic mean of ten children seen at each of the ten selected health facility of the eight districts per step (and therefore 100 children per district and 800 children per step), the trial would provide 90% power to detect an increase in any of the primary outcome from 25 to 33% %. With a harmonic mean of only four children seen per facility at each step, the trial would have 98% power to detect an increase from 25 to 40% [21].

### Data collection

Data collection was conducted by two teams, each comprising two trained nurses. At each step, all ten selected primary facilities in each of the eight districts, were visited once for data collection. Data were collected for all consultations of children aged 2 months to 5 years old occurring during the research team’s visit to the facility. Each visit lasted 2 days or less if the required minimum sample size of children observed per facility was achieved. At each step, the newest intervention district was visited last to maximise the chances that HCWs had learnt how to use the new technology. Each visit was notified, by the data collection team, to the facility the day before the visit.

One independent trained nurse observed the consultation and recorded, using a structured and pre-tested observation form programmed into a tablet, the HCW’s clinical practices, illness classifications and prescriptions given to the child. Observations were passive, and the observer never intervened during the consultation. Validation data were collected by the second independent trained nurse, who conducted a repeat consultation with the child, using the eCDSS. These validation data were intended to provide a “gold standard” classification for each child. When there were discrepancies between the HCW and the validation nurse, the final management of the child was agreed by discussion between the two of them.

In addition, at each visit, a shortened version of the WHO Service Availability and Readiness Assessment (SARA) questionnaire [23] was completed to document the availability of essential medicines and equipment required by IMCI guidelines.

The four nurses recruited for data collection had previously been trained in IMCI by the MoH. The two nurses responsible for observation of consultations had at least 5 years of experience working in a health centre. The two validation nurses had at least 10 years of experience working in a health centre and were also IMCI trainers. In addition, all underwent 2 weeks of training, provided by the main investigators, on the study methods and tools prior to the trial, and benefited from two refresher trainings, provided by Tdh, on IMCI and the eCDSS during the trial.

### Outcomes

The evaluation focussed on the adherence to IMCI charts designed for new consultations of children aged 2 months to 5 years old to assess, classify and treat danger signs, cough/difficult breathing, diarrhoea, fever and nutritional status.The evaluation did not consider IMCI charts designed for children who return after an intial consultation. We excluded charts related to HIV and ear problems due to their very low prevalence during the trial period (across all steps and according to the validation nurses, only 0.9% of children classified with HIV infection, and 2.7% of children classified with ear problems). We also excluded the charts related to vitamin A supplementation and vaccination as coverage was high in Burkina Faso. Upon the advice of the trial’s scientific advisory committee, for anaemia, only adherence to the clinical assessment task was evaluated due to the difficulty of assessing anaemia reliably when laboratory testing was locally unavailable.

Primary and secondary outcomes are defined in the Additional file 1. Briefly, the primary outcomes included: 1. overall adherence to clinical assessment tasks; 2. overall correct classification ignoring the severity of the classifications (upon the advice of the trial’s scientific advisory committee); and 3. overall correct prescription according to HCWs’ classifications. The secondary outcomes included: 1. adherence to assessment of danger signs; 2. correct identification of at least one danger sign; 3. overall correct classification accounting for the severity of the classifications; 4. overall correct prescription according to validation nurses’ classifications; 5 & 6. overall correct referral or hospitalisation according to HCWs’ assessment and to validation nurses’ assessment; and 7. overall correct treatment counselling.

Other reported outcomes are: sensitivity and specificity of the HCWs’ classifications; over-prescription of antibiotics and antimalarials; overall availability index of essential oral medicines and equipment (Additional file 2).

### Analyses

Analyses were performed using Stata version 14. Analysis included all new consultations of children aged 2 months to 5 years old and excluded children who return after an intial consultation for a follow-up consultation. Primary analyses included “contaminated” control districts as control districts based on the intention-to-treat (ITT) principle.

Secondary analyses excluded these districts for the period when they were contaminated.

Descriptiive analyses were performed using individual-level data and point estimates and confidence intervals for all outcomes were computed accounting for the clustering of observations within districts and facilities using the svy family of commands in Stata.

Comparisons between trial arms and statistical tests to investigate evidence of an intervention effect were performed on cluster/step-level summaries as recommended by Hayes and Moulton [24] for trials with fewer than about 15 clusters per arm to account for the clustered nature of the data. A “vertical” stepped wedge analysis was performed with permutation test using the swpermute command in Stata [25]. This approach analyses each step as a parallel arm trial or, in other words, computes, for each step, one cluster summary per district and one effect estimate and then combines these step-level effect estimates into a weighted average (with the weights proportional to the harmonic mean of the number of clusters in each arm and step). This approach, recommended by Thomson et al. [26], preserves the randomisation and accounts for secular trends. “Horizontal” comparisons, i.e. comparison within a cluster over time (which are non-randomised), do not contribute to the analysis. Applied to our design, across the six steps and the eight clusters, 46 cluster/step summaries were computed (two cluster/step-level summaries were excluded from the analysis due to data lost in two districts at step 6 and 7 respectively) giving six effect estimates which were then combined into a weighted average for each of our outcome.

The above approach was used for all primary and secondaty outomes with the exception of correct identification of at least one danger sign and overall correct referral/hospitalisation. Given the very small number of children with danger signs or severe classifications warranting referral/hospitalisation who contributed to these two outcomes, Fisher’s exact test, performed on individual level data and ignoring clustering, was used to test for an intervention effect.

Statistical tests to investigate evidence of a difference between trial arms were only performed on the primary and secondary outcomes to reduce the problem of multiple testing. No formal adjustment was made for multiple testing. Because our ten endpoints are not all independent to each other, applying the Bonferronni correction would be overly conservative (as it assumes that all hypotheses being tested are independent of each other).

## Results

**Fig. 3:**
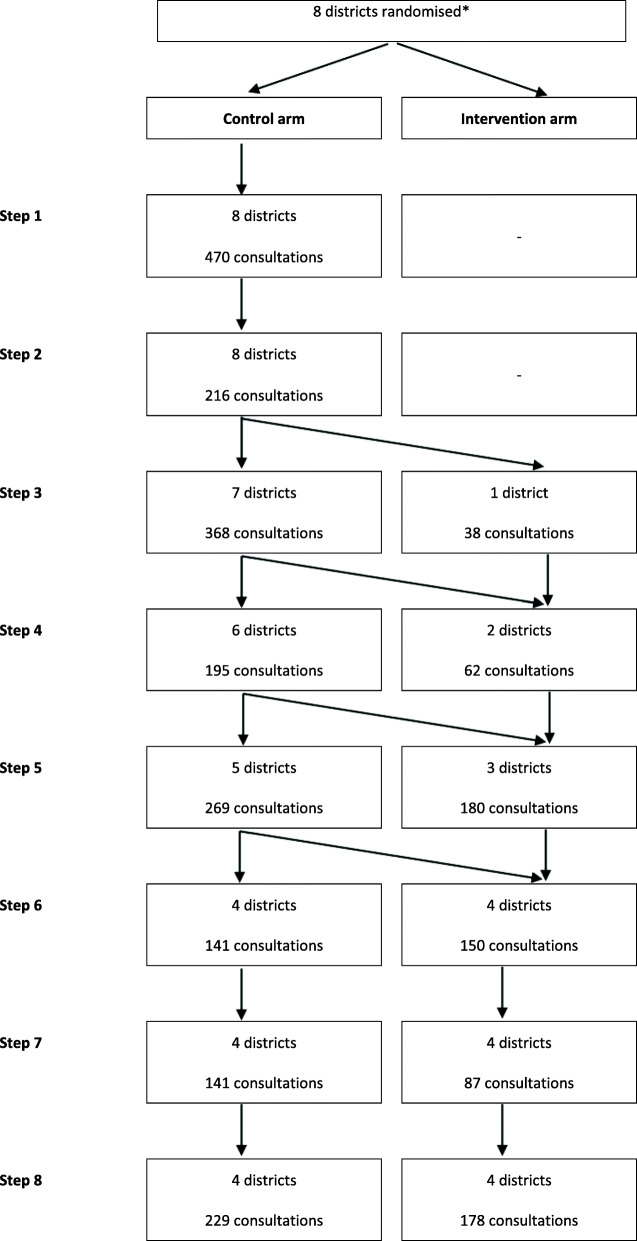
Trial flow diagram (number of consultations of children aged 2 to 60 months). * Eight districts randomised but only 4 actually received the IeDA intervention

After excluding 189 follow-up consultations, data were recorded for 2724 new consultations of children aged 2 months to 5 years old: 686 consultations at baseline, 1343 consultations in control districts and 695 consultations in intervention districts (Fig. 3, Additional file 4).

About half of consultations at baseline and in the control and intervention districts (46%, 317/686, 49%, 658/1343, and 48%, 335/695 respectively) were conducted in districts where the concurrent PBF intervention was implemented.

While the IMCI paper-form was used for 70% (479/686) and 68% (918/1343) of the consultations at baseline and in control clusters respectively, the eCDSS was used in nearly all consultations (97%, 674/694) in intervention clusters. The occasional use of the eCDSS at baseline (1%, 8/686) or in the control districts (9%, 120/1343) reflects instances of early roll-out of the eCDSS prior to training.

Gender and age distributions were similar at baseline and by trial arm (Table 1). Based on validation nurses’ assessment, the most common classification given to children was malaria (between 53 and 69% of children across baseline and trial arms) (Table 2). Other common classifications included: diarrhoea with no dehydration (about 27%) and pneumonia (between 16 and 27%). About 45% of children had one classification only and between 33 and 48% had two or more classifications (Table 3).

**Table 1:** Child’s gender and age at baseline and by trial arm

		Baseline (*N* = 686)	Control arm (*N* = 1343)	Intervention arm (*N* = 695)
		%	%	%
Gender	Females	45.3	44.4	45.9
	Males	54.7	55.6	54.1
	Total	100.0	100.0	100.0
Age (months)	2–11	31.8	29.0	31.7
	12–23	28.6	30.9	27.8
	24–35	18.4	17.9	18.9
	36–47	11.4	12.2	12.4
	48–60	9.9	10.0	9.4
	Total	100.0	100.0	100.0

**Table 2:** Validation nurses’ classifications at baseline and by trial arm

	Baseline (*N* = 686)	Control arm (*N* = 1343)	Intervention arm (*N* = 695)
Classification	%	%	%
Severe pneumonia or very severe disease	2.2	0.7	0.6
Pneumonia	27.3	16.2	24.3
Severe dehydration	0.7	0.1	0.0
Dehydration	0.7	0.2	0.4
Diarrhoea with no dehydration	25.7	25.5	27.2
Severe persistent diarrhoea	0.0	0.0	0.0
Persistent diarrhoea	0.0	0.0	0.0
Dysentery	1.8	2.0	1.7
Severe malaria or severe febrile illness	3.4	1.8	2.5
Malaria	69.4	54.7	52.7
Severe and complicated measles	0.0	0.0	0.0
Measles with eyes or mouth complications	0.0	0.0	0.0
Measles	0.0	0.0	0.0
Severe anaemia	0.4	0.2	0.1
Anaemia	12.5	6.6	4.5
Severe acute malnutrition	4.6	4.9	4.0
Moderate acute malnutrition	10.8	11.4	16.2

**Table 3:** Number of classifications according to validation nurses at baseline and by trial arm

Number of classifications	Baseline (N = 686)	Control arm (N = 1343)	Intervention arm (N = 695)
	%	%	%
0	10.5	21.3	17.3
1	41.3	45.5	44.6
2	32.7	24.7	28.4
3	11.8	6.9	8.5
4	3.2	1.5	1.2
5	0.6	0.2	0.1
Total	100.0	100.0	100.0

### Adherence to clinical assessment

Across the six IMCI charts, the average percentage of tasks completed by the HCWs was 48% at baseline, 54% in the control districts and 79% in the intervention districts with evidence for a difference between trial arms (cluster-level mean difference = 30%; *P*-value = 0.002) (Table 4). For all IMCI charts, HCWs in the intervention districts completed more of the recommended tasks compared to HCWs in the control districts (Table 5). In particular, more of the recommended tasks were completed for assessing danger signs: 95% versus 34% in the intervention and control districts respectively (cluster-level mean difference = 71%; *P*-value = 0.002) (Table 4).

**Table 4:** Primary and secondary outcomes

**Adherence to clinical assessment**	Baseline				Control arm				Intervention arm				Cluster-level mean difference between arms	*P*-value*
	N	%	95%CI		N	%	95%CI		N	%	95%CI			
Overall adherence (13 to 33 tasks)	686	**48.0**	44.3	51.6	1343	**54.3**	50.6	58.0	695	**79.3**	72.7	85.9	**29.9**	0.002
Adherence to danger signs’ assessment (3 tasks)	686	**18.4**	12.0	24.9	1343	**34.2**	25.5	42.9	695	**95.2**	90.0	99.9	**71.2**	0.002
**Identification of at least one danger sign** (proportion of children correctly identified with at least one danger sign)	Baseline				Control arm				Intervention arm				Individual-level difference between arms	*P*-value**
	N^a^	%	95%CI		N^a^	%	95%CI		N^a^	%	95%CI			
	24	**66.7**	47.2	81.7	25	**56.0**	30.8	78.4	16	**75.0**	50.5	89.8	**19.0**	0.322
**Overall correct classification** (proportion of children correctly classified with x given classifications)	Baseline				Control arm				Intervention arm				Cluster-level mean difference between arms	*P*-value*
	N^b^	%	95%CI		N^b^	%	95%CI		N^b^	%	95%CI			
Accounting for the severity of classifications	609	**70.6**	63.7	76.7	1049	**69.8**	66.0	73.4	572	**74.7**	66.9	81.1	**9.1**	0.038
Ignoring the severity of the classifications	609	**75.0**	68.0	81.0	1049	**73.1**	68.8	77.0	572	**78.7**	72.9	83.5	**10.1**	0.004
**Overall correct prescription** (proportion of children who received at least all the recommended prescriptions)	Baseline				Control arm				Intervention arm				Cluster-level mean difference between arms	*P*-value*
	N^c^	%	95%CI		N^c^	%	95%CI		N^c^	%	95%CI			
According to the HCWs’ classifications	614	**75.7**	68.0	82.1	1074	**77.8**	72.5	82.4	567	**77.1**	71.6	81.8	**−1.1**	0.788
According to the validation nurses’ classifications	610	**65.3**	59.8	70.4	1049	**66.1**	60.7	71.0	572	**68.5**	58.8	76.9	**6.7**	0.226
**Overall correct referral/hospitalisation** (proportion of children in need of referral/hospitalisation who were actually referred/hospitalised)	Baseline				Control arm				Intervention arm				Individual-level difference between arms	*P*-value**
	N^d^	%	95%CI		N^d^	%	95%CI		N^d^	%	95%CI			
According to the HCWs’ classifications	35	**60.0**	47.7	71.1	42	**52.4**	23.7	79.6	41	**61.0**	21.5	89.9	**8.6**	0.509
According to the validation nurses’ classifications	29	**55.2**	36.0	72.9	32	**53.1**	36.5	69.1	22	**68.2**	47.8	83.4	**15.1**	0.398
**Overall correct treatment counselling** (proportion of caretakers who received information on home-based prescription)	Baseline				Control arm				Intervention arm				Cluster-level mean difference between arms	*P*-value*
	N^e^	%	95%CI		N^e^	%	95%CI		N^e^	%	95%CI			
	612	**77.3**	67.5	84.8	1143	**91.5**	88.8	93.6	576	**87.9**	77.9	93.7	**−4.1**	0.355

* t test on cluster-level summaries & accounting for the stepped wedge design; ** Fisher’s exact test on individual-level data & ignoring clustering

^a^Number of children identified, by the validation nurses, with a given danger sign

^b^ Number of children classified, by the validation nurses, with x given classification

^c^Number of children classified, by the HCWs or by the validation nurses, with x given classification

^d^Number of children identified, by the HCWs or the validation nurses, with at least one danger sign or a classification requiring referral/hospitalisation

e Number of children who were prescribed, by the HCWs, x given treatment (regardless of the classification)

**Table 5:** Adherence to clinical assessment by IMCI chart

IMCI chart	Task: Questions to address to the mother or examinations to perform	Baseline				Control arm				Intervention arm			
		N	%	95%CI		N	%	95%CI		N	%	95%CI	
Danger signs ^b^	Ask if the child is able to drink/breastfeed	686	**15.9**	9.2	26.1	1343	**28.7**	20.4	38.8	695	**94.5**	75.1	99.0
	Ask if the child vomits everything	686	**15.7**	8.6	27.0	1342	**40.8**	33.0	49.0	695	**95.8**	81.2	99.2
	Ask about recent convulsions ^a^	686	**23.6**	15.0	35.1	1342	**33.1**	23.3	44.6	695	**95.3**	90.3	97.7
	**Adherence index (3 tasks)**	686	**18.4**	12.0	24.9	1343	**34.2**	25.5	42.9	695	**95.2**	90.0	99.9
Cough/difficult breathing	Ask about cough ^a^	686	**94.8**	90.4	97.2	1342	**94.3**	87.2	97.6	695	**99.4**	97.4	99.9
	Ask about difficult breathing ^a^	686	**2.8**	1.6	4.8	1295	**7.9**	3.1	18.6	605	**11.4**	4.0	28.5
	Ask for duration (if cough/difficult breathing)	188	**93.1**	85.4	96.9	450	**82.2**	74.4	88.1	324	**96.9**	87.5	99.3
	Count number of breaths per minute (if cough/difficult breathing)	317	**54.9**	40.6	68.4	554	**44.8**	34.0	56.1	340	**88.5**	79.2	94.0
	Look for chest indrawing (if cough/difficult breathing)	318	**47.8**	37.5	58.2	554	**41.2**	28.9	54.7	340	**82.4**	75.0	87.9
	Listen for stridor or “wheeze” breathing (if cough/difficult breathing)	318	**27.0**	17.9	38.7	554	**17.2**	10.8	26.1	339	**51.6**	18.8	83.1
	**Adherence index (2 to 6 tasks)**	686	**48.3**	44.4	52.2	1342	**50.0**	45.8	54.3	695	**67.8**	55.0	80.7
Diarrhoea ^c^	Ask about diarrhoea ^a^	686	**94.8**	87.4	97.9	1343	**92.3**	85.1	96.2	695	**98.7**	94.8	99.7
	Ask for duration (if diarrhoea)	152	**92.1**	86.2	95.6	340	**87.1**	80.0	91.9	178	**96.6**	90.5	98.9
	Ask for blood in the stool (if diarrhoea) ^a^	151	**53.0**	45.3	60.5	346	**58.4**	45.3	70.4	178	**89.9**	81.3	94.8
	Offer water to the child (if diarrhoea)	151	**9.9**	3.1	27.5	347	**6.6**	3.6	11.8	178	**41.6**	21.7	64.6
	Pinch the skin of the abdomen (if diarrhoea)	98	**46.9**	35.0	59.3	285	**47.0**	26.1	69.0	172	**76.7**	65.7	85.0
	**Adherence index (1 to 5 tasks)**	686	**86.2**	81.3	91.2	1343	**82.1**	74.9	89.2	695	**93.8**	91.6	96.1
Fever or history of fever ^d^	Ask about current fever ^a^	686	**88.2**	72.7	95.4	1343	**96.9**	92.1	98.8	694	**100.0**	–	–
	Ask about history of fever ^a^	686	**23.5**	14.0	36.6	1319	**75.1**	51.2	89.7	654	**72.9**	42.8	90.6
	Ask for duration (if fever)	543	**91.7**	87.0	94.8	1027	**88.4**	84.5	91.4	575	**96.7**	95.0	97.8
	Ask if the child had fever every day (if fever > 7 days)	8	**25.0**	2.8	79.5	10	**40.0**	13.6	73.9	4	**75.0**	17.4	97.7
	Ask if urine are dark or not abundant (if fever)	544	**10.9**	6.8	16.9	1081	**19.8**	13.9	27.5	581	**81.2**	67.8	89.9
	Ask about abnormal bleeding (if fever)	544	**6.3**	3.0	12.4	1081	**14.7**	7.5	26.8	581	**75.7**	59.0	87.1
	Ask about history of measles in the past 3 months (if fever)	544	**10.9**	6.4	17.8	1081	**17.7**	13.2	23.2	581	**65.8**	29.7	89.7
	Take the temperature (if fever)	544	**99.8**	98.4	99.9	1082	**99.1**	98.5	99.4	586	**99.7**	98.9	99.9
	Perform a RDT (if fever)	544	**74.3**	53.8	87.7	1082	**86.7**	79.5	91.6	584	**93.7**	88.6	96.6
	Look for neck stiffness (if fever)	544	**16.2**	7.6	31.3	1081	**23.0**	15.2	33.3	582	**47.6**	37.8	57.6
	Take the pulse (if fever)	544	**2.4**	0.5	9.9	1075	**8.2**	2.7	22.3	582	**1.6**	0.4	5.6
	Look for cold hands or feet (if fever)	544	**1.3**	0.4	4.5	1078	**1.2**	0.5	2.7	578	**33.7**	11.0	67.7
	Look for jaundice or redness in the eyes (if fever)	544	**82.5**	70.8	90.2	1081	**83.0**	73.3	89.6	584	**94.0**	86.9	97.4
	Look for general rash (if fever)	544	**14.9**	7.4	27.6	1082	**14.1**	9.3	20.6	583	**34.3**	21.0	50.6
	**Adherence index (2 to 14 tasks)**	686	**40.3**	38.1	42.5	1343	**52.5**	47.8	57.2	694	**71.5**	65.6	77.4
Anaemia	Look for palmar pallor	686	**59.9**	44.2	73.8	1341	**51.5**	42.6	60.4	692	**92.2**	78.4	97.5
Nutritional status ^e^	Measure height	686	**88.3**	61.8	97.3	1343	**90.4**	79.1	95.9	695	**99.6**	98.9	99.8
	Weigh the child	686	**98.5**	94.3	99.6	1343	**98.4**	93.7	99.6	695	**99.1**	97.5	99.7
	Measure MUAC	686	**84.3**	70.4	92.3	1342	**86.1**	82.2	89.2	692	**88.4**	83.5	92.1
	Look for feet oedema	686	**23.5**	14.3	36.1	1342	**35.1**	22.8	49.8	692	**86.6**	55.0	97.1
	Offer RUTF (if age > 5 months & MUAC < 115 mm & no danger sign or severe classification)	17	**0.0**	–	–	34	**8.8**	1.9	32.8	16	**43.8**	20.3	70.4
	**Adherence index (4 to 5 tasks)**	686	**73.2**	64.5	81.9	1343	**77.1**	71.6	82.5	695	**93.2**	87.3	99.1

^a^ Or reported by the caretakers spontaneously when the consultation started or when asked the reason for consulting; ^b^ Tasks recommended by the IMCI guidelines but not observable during consultations: Observe if the child convulses, observe if the child is lethargic/unconscious; ^c^ Tasks recommended by the IMCI guidelines but not observable during consultations: Observe if the child is lethargic/unconscious (if diarrhoea), observe if the child is restless/irritated (if diarrhoea), look for sunken eyes (if diarrhoea); ^d^ Tasks recommended by the IMCI guidelines but not observable during consultations: Look for running nose (if fever); ^e^ Tasks recommended by the IMCI guidelines but not observable during consultations: Look for severe and visible weight loss

### Identification of danger signs

The proportion of children correctly identified, by the HCWs, with at least one danger sign was 67% (16/24) at baseline and 56% (14/25) in the control districts. It appeared to be somewhat higher (75%, 12/16) in the intervention districts but the small number of children with danger signs preclude firm conclusion (cluster-level mean difference = 19%; *P*-value = 0.322) (Table 4).

### Classification

Ignoring the severity of the classifications, the proportion of children for whom the validation nurses and the HCWs recorded the same classifications was 75% (457/609) at baseline, 73% (767/1049) in the control districts and 79% (450/572) in the intervention districts with evidence for a difference between trial arms (cluster-level mean difference = 10%; P-value = 0.004) (Table 4). Accounting for the severity of the classifications slightly lowered the proportions of correct classifications (cluster-level mean difference = 9%; P-value = 0.038) (Table 4).

By IMCI chart, HCWs in the intervention districts correctly classified children having diarrhoea with no dehydration, dysentery and acute malnutrition (severe or moderate) more often than those in the control districts (Table 6). Although based on a small number of children, HCWs in intervention districts also appeared to correctly classify children with severe malaria or severe febrile illness more often than those in control districts.

**Table 6:** Sensitivity of the HCW’s classification: Proportion of children correctly classified in a given classification

	Baseline				Control arm				Intervention arm			
Classification	N^a^	%	95%CI		N^a^	%	95%CI		N^a^	%	95%CI	
Severe pneumonia or very severe disease	14	**14.3**	2.5	51.7	10	**20.0**	1.3	82.6	4	**25.0**	–	–
Pneumonia	185	**75.7**	66.6	82.9	218	**71.6**	65.6	76.9	169	**76.3**	46.8	92.2
**Pneumonia ignoring severity**	199	**75.9**	67.0	83.0	228	**73.3**	65.8	79.5	173	**78.6**	50.5	93.0
Severe dehydration	5	**60.0**	20.1	90.0	1	**0.0**	–	–	0	**–**	–	–
Dehydration	5	**80.0**	17.1	98.7	2	**50.0**	0.6	99.4	3	**66.7**	1.1	99.7
Diarrhoea with no dehydration	175	**58.3**	44.5	70.9	343	**64.7**	51.9	75.7	189	**74.6**	62.9	83.6
Severe persistent diarrhoea	0	**–**	–	–	0	**–**	–	–	0	**–**	–	–
Persistent diarrhoea	0	**–**	–	–	0	**–**	–	–	0	**–**	–	–
**Diarrhoea ignoring severity**	185	**61.1**	48.1	72.7	346	**65.9**	52.6	77.1	192	**76.6**	64.8	85.3
**Dysentery**	12	**41.7**	22.9	63.2	27	**44.4**	29.3	60.6	12	**83.3**	17.8	99.1
Severe malaria or severe febrile illness	23	**60.9**	37.7	80.0	24	**62.5**	36.1	83.1	17	**82.4**	53.2	95.0
Malaria	475	**93.9**	90.9	96.0	734	**92.0**	87.6	94.9	366	**91.0**	87.7	93.4
**Malaria ignoring severity**	498	**95.0**	91.9	96.9	758	**93.1**	90.0	95.3	383	**93.0**	87.5	96.1
Severe acute malnutrition	22	**81.8**	50.0	95.3	45	**57.8**	37.6	75.6	23	**91.3**	67.6	98.1
Moderate acute malnutrition	56	**46.4**	29.9	63.8	120	**41.7**	31.6	52.5	95	**62.1**	52.2	71.1
**Malnutrition ignoring severity**	78	**68.0**	52.8	80.0	165	**55.2**	45.5	64.4	118	**75.4**	63.1	84.6

^a^ Number of children classified, by the validation nurses, with a given classification

HCWs in the intervention districts were also less likely to wrongly diagnose pneumonia as being present when it was not: 7% (38/521) versus 19% (209/1113) (Table 7). For other conditions, false positive diagnoses were rare (< 5%) in both arms.

**Table 7:** Specificity of the HCW’s classification: Proportion of children correctly not classified in a given classification

	Baseline				Control arm				Intervention arm			
Classification	N^a^	%	95%CI		N^a^	%	95%CI		N^a^	%	95%CI	
Severe pneumonia or very severe disease	640	**99.8**	98.6	99.9	1331	**99.5**	98.4	99.8	690	**98.8**	96.3	99.6
Pneumonia	469	**82.5**	72.0	89.6	1123	**81.2**	72.5	87.6	525	**93.1**	78.4	98.1
**Pneumonia ignoring severity**	455	**83.7**	73.4	90.6	1113	**81.2**	72.9	87.4	521	**92.7**	76.4	98.0
Severe dehydration	675	**100.0**	–	–	1342	**99.9**	99.4	99.9	695	**100.0**	–	–
Dehydration	681	**99.7**	98.3	99.9	1341	**99.6**	98.5	99.9	692	**99.3**	98.6	99.6
Diarrhoea with no dehydration	505	**97.0**	94.9	98.3	1000	**94.7**	89.0	97.5	506	**98.8**	97.4	99.5
Severe persistent diarrhoea	680	**99.9**	98.1	99.9	1343	**100.0**	–	–	695	**100.0**	–	–
Persistent diarrhoea	680	**99.9**	98.1	99.9	1343	**100.0**	–	–	695	**99.9**	99.1	99.9
**Diarrhoea ignoring severity**	495	**97.0**	94.8	98.2	997	**94.5**	88.8	97.4	503	**98.4**	95.6	99.4
**Dysentery**	667	**97.5**	93.0	99.0	1316	**97.5**	95.0	98.8	683	**99.6**	98.9	99.8
Severe malaria or severe febrile illness	660	**98.8**	97.7	99.4	1319	**99.2**	96.1	99.8	678	**98.5**	96.1	99.5
Malaria	208	**90.9**	83.4	95.2	609	**95.2**	90.7	97.6	329	**95.7**	91.9	97.8
**Malaria ignoring severity**	185	**92.4**	85.6	96.2	585	**95.9**	92.3	97.9	312	**95.2**	91.8	97.2
Severe acute malnutrition	563	**97.9**	95.4	99.0	1100	**98.9**	98.4	99.3	589	**98.5**	96.7	99.3
Moderate acute malnutrition	529	**93.6**	89.7	96.0	1025	**96.8**	94.0	98.3	517	**96.5**	94.3	97.9
**Malnutrition ignoring severity**	507	**92.7**	89.2	95.2	980	**96.9**	94.0	98.5	494	**96.4**	94.1	97.8

^a^ Number of children not classified, by the validation nurses, with a given classification

### Prescription

Overall, the proportion of children who received all the recommended prescriptions in accordance with the HCWs’ classifications was 76% (465/614) at baseline, 78% (836/1074) in the control districts and 77% (437/567) in the intervention districts with no evidence for a difference between trial arms (cluster-level mean difference = − 1%; *P*-value = 0.788) (Table 4). According to the validation nurses’ classifications, these proportions were 65% (398/610) at baseline, 66% (693/1049) in the control districts and 69% (392/572) in the intervention districts (cluster-level mean difference = 7%; *P*-value = 0.226).

By IMCI chart, correct prescriptions for dysentery were much more common in the intervention districts than in the control districts, as were correct prescriptions for acute malnutrition (severe without complications or moderate) and severe malaria or severe febrile illness, although still infrequent (Tables 8 and 9). Correct prescriptions for diarrhoea with no dehydration were also higher in the intervention districts compared to the control districts (Table 9).

**Table 8:** Correct prescription according to the HCWs’ classifications: Proportion of children who received at least all the recommended prescriptions

	Baseline				Control arm				Intervention arm			
Classification	N^b^	%	95%CI		N^b^	%	95%CI		N^b^	%	95%CI	
Severe pneumonia or very severe disease	3	**33.3**	2.2	91.7	9	**44.4**	9.7	85.7	9	**55.6**	25.5	82.0
Pneumonia	222	**93.2**	89.7	95.6	367	**94.8**	86.7	98.1	165	**95.2**	92.8	96.8
**All classifications related to pneumonia**	225	**92.4**	88.1	95.3	376	**93.6**	86.2	97.2	174	**93.1**	88.8	95.8
Severe dehydration with another severe classification	3	**33.3**	2.2	91.7	1	**0.0**	–	–	0	**–**	–	–
Severe dehydration without another severe classification	0	**–**	–	–	0	**–**	–	–	0	**–**	–	–
Dehydration with other severe classification	3	**66.7**	8.3	97.8	2	**50.0**	0.6	99.4	2	**100.0**	–	–
Dehydration without other severe classification	3	**100.0**	–	–	5	**80.0**	7.1	99.5	5	**100.0**	–	–
Diarrhoea with no dehydration	117	**70.1**	52.7	83.1	275	**84.0**	79.4	87.7	147	**88.4**	73.3	95.5
Severe persistent diarrhoea	1	**100.0**	–	–	0	**–**	–	–	0	**–**	–	–
Persistent diarrhoea	1	**0.0**	–	–	0	**–**	–	–	1	**0.0**	–	–
**All classifications related to diarrhoea**	128	**69.5**	53.3	82.0	283	**83.4**	79.3	86.8	155	**88.4**	72.8	95.6
**Dysentery**	22	**0.0**	–	–	45	**11.1**	2.3	39.4	13	**69.2**	34.7	90.5
Severe malaria or severe febrile illness	22	**9.1**	1.6	38.6	26	**7.7**	1.3	34.6	24	**33.3**	7.9	74.5
Malaria	465	**98.9**	97.4	99.6	704	**98.9**	97.3	99.5	347	**98.3**	96.1	99.2
**All classifications related to malaria**	487	**94.9**	91.9	96.8	730	**95.6**	93.1	97.2	371	**94.1**	84.9	97.8
Severe acute malnutrition with complications^a^	3	**0.0**	–	–	5	**40.0**	7.2	85.1	0	**–**	–	–
Severe acute malnutrition without complications	25	**32.0**	17.3	51.5	35	**17.1**	5.0	45.0	30	**40.0**	30.7	50.1
Moderate acute malnutrition	64	**12.5**	7.0	21.2	84	**1.2**	0.1	11.1	82	**8.5**	5.1	14.0
**All classifications related to malnutrition**	95	**16.8**	10.3	26.3	124	**7.3**	4.2	12.3	112	**17.0**	10.1	27.0

^a^ Any danger sign or other severe classification

^b^ Number of children classified, by the HCWs, with a given classification

**Table 9:** Correct prescription according to the validation nurses’ classifications: Proportion of children who received at least all the recommended prescriptions

	Baseline				Control arm				Intervention arm			
Classification	N^b^	%	95%CI		N^b^	%	95%CI		N^b^	%	95%CI	
Severe pneumonia or very severe disease	15	**6.7**	0.7	43.6	10	**0.0**	–	–	4	**0.0**	–	–
Pneumonia	187	**81.8**	74.1	87.6	218	**78.9**	70.8	85.2	169	**81.1**	61.5	92.0
**All classifications related to pneumonia**	202	**76.2**	69.1	82.2	228	**75.4**	65.3	83.4	173	**79.2**	55.2	92.1
Severe dehydration with another severe classification	5	**20.0**	2.7	69.1	1	**0.0**	–	–	0	**–**	–	–
Severe dehydration without another severe classification	0	**–**	–	–	0	**–**	–	–	0	**–**	–	–
Dehydration with other severe classification	1	**100.0**	–	–	2	**50.0**	–	–	0	**–**	–	–
Dehydration without other severe classification	4	**50.0**	4.3	95.7	0	**–**	–	–	3	**66.7**	–	–
Diarrhoea with no dehydration	176	**50.0**	40.5	59.5	343	**65.6**	57.5	72.9	189	**76.7**	52.9	90.6
Severe persistent diarrhoea	0	**–**	–	–	0	**–**	–	–	0	**–**	–	–
Persistent diarrhoea	0	**–**	–	–	0	**–**	–	–	0	**–**	–	–
**All classifications related to diarrhoea**	186	**49.5**	39.8	59.2	346	**65.3**	57.5	72.4	192	**76.6**	52.1	90.8
**Dysentery**	12	**8.3**	0.7	54.0	27	**11.1**	2.1	42.4	12	**75.0**	29.2	95.6
Severe malaria or severe febrile illness	23	**8.7**	1.4	38.7	24	**0.0**	–	–	17	**29.4**	4.5	78.7
Malaria	476	**94.5**	91.7	96.4	734	**94.1**	90.3	96.5	366	**95.4**	92.1	97.3
**All classifications related to malaria**	499	**90.6**	87.1	93.2	758	**91.2**	87.0	94.1	383	**92.4**	82.4	97.0
Severe acute malnutrition with complications^a^	3	**0.0**	–	–	3	**33.3**	0.1	99.9	0	–	–	–
Severe acute malnutrition without complications	19	**15.8**	5.5	37.8	42	**14.3**	4.1	39.6	23	**43.5**	33.0	54.6
Moderate acute malnutrition	57	**8.8**	4.6	16.2	121	**2.5**	0.5	11.2	95	**8.4**	5.4	12.9
**All classifications related to malnutrition**	79	**10.1**	5.3	18.6	166	**6.0**	3.9	9.2	118	**15.3**	11.1	20.6

^a^ Any danger sign or other severe classification

^b^ Number of children classified, by the validation nurses, with a given classification

### Over-prescription

According to the HCWs’ classifications, the proportion of children who were not in need of an antibiotic but who were actually prescribed one was 11% (77/681) at baseline, 14% (187/1341) in the control districts and 8% (56/694) in the intervention districts (Table 10). According to validation nurses’ classifications, these proportions were 18% (123/668) at baseline, 23% (289/1252) in the control districts and 10% (69/676) in the intervention districts (Table 11). With respect to antimalarials, a Rapid Diagnostic Test (RDT) was performed for about 90% of febrile children in both arms (Table 5) and over-prescription was low and similar at baseline and between trial arms: around 2 to 4%.

**Table 10:** Over-prescription according to the HCWs’ classifications: Proportion of children who were not in need of a given medicine but who were actually prescribed it

	Baseline (*N* = 686)			Control arm (*N* = 1343)			Intervention arm (*N* = 695)		
Medicines	%	95%CI		%	95%CI		%	95%CI	
Ampicillin injectable	**0.0**	–	–	**0.1**	–	–	**0.3**	0.0	1.8
Gentamycin injectable	**0.0**	–	–	**0.0**	–	–	**0.1**	–	–
Cotrimoxazole	**6.1**	4.0	9.3	**6.7**	4.5	9.8	**1.9**	0.8	4.3
Amoxicillin	**4.0**	2.2	7.1	**5.8**	3.3	10.0	**5.0**	3.3	7.6
Ciprofloxacin	**0.3**	–	–	**0.0**	–	–	**0.3**	–	–
Metronidazole	**0.9**	0.2	3.8	**2.0**	1.4	2.8	**0.6**	0.1	2.2
**All antibiotics**	**11.3**	8.3	15.3	**13.9**	9.2	20.5	**8.1**	5.2	12.4
Artesunate or Artemether injectable	**0.4**	0.1	1.5	**0.1**	–	–	**0.1**	–	–
Quinine injectable	**0.0**	–	–	**0.0**	–	–	**0.0**	–	–
Artemisinin-based Combinnation Therapy (ACT)	**1.3**	0.5	3.8	**1.7**	0.7	4.0	**2.6**	1.0	6.8
**All antimalarials**	**1.8**	0.8	3.8	**1.8**	0.8	4.0	**2.7**	1.0	7.2

*ACT* Artesunate + Amodiaquine or Artemether/ Lumefantrine

**Table 11:** Over-prescription according to the validation nurses’ classifications: Proportion of children who were not in need of a given medicine but who were actually prescribed it

	Baseline (N = 686)			Control arm (*N* = 1343)			Intervention arm (*N* = 695)		
Medicines	%	95%CI		%	95%CI		%	95%CI	
Ampicillin injectable	**0.6**	0.2	1.4	**0.3**	0.1	1.5	**1.0**	0.3	2.9
Gentamycin injectable	**0.0**	–	–	**0.3**	0.1	1.6	**0.9**	0.3	2.2
Cotrimoxazole	**10.4**	6.0	17.3	**8.6**	5.4	13.5	**1.3**	0.4	3.7
Amoxicillin	**5.3**	3.1	8.9	**10.7**	5.7	19.2	**6.5**	3.2	12.5
Ciprofloxacin	**0.2**	–	1.5	**0.2**	–	0.7	**0.3**	0.1	0.6
Metronidazole	**1.9**	0.7	4.8	**3.0**	2.0	4.4	**1.0**	0.3	2.9
**All antibiotics**	**18.4**	11.8	27.5	**23.1**	16.3	31.7	**10.2**	4.7	20.7
Artesunate or Artemether injectable	**0.7**	0.3	2.0	**0.6**	0.1	4.1	**0.7**	0.3	1.7
Quinine injectable	**0.4**	0.1	1.5	**0.2**	–	1.4	**0.7**	0.3	1.7
Artemisinin-based Combinnation Therapy (ACT)	**2.8**	2.0	3.8	**2.1**	0.8	5.6	**1.6**	0.5	4.5
**All antimalarials**	**3.9**	2.6	6.0	**2.8**	1.2	6.3	**3.0**	1.2	7.4

*ACT* Artesunate + Amodiaquine or Artemether/ Lumefantrine

### Referral/ hospitalisation

Overall, the proportion of children in need of referral or hospitalisation according to the HCWs’ assessment who were actually referred or hospitalised by the HCWs was 60% (21/35) at baseline, 52% (22/42) in the control districts and 61% (25/41) in the intervention districts with no evidence for a difference between trial arms (individual-level mean difference = 9%; *P*-value = 0.509) (Table 4). According to the validation nurses’ assessment, these proportions were 55% (16/29) at baseline, 53% (17/32) in the control districts and 68% (15/22) in the intervention districts (individual-level mean difference = 15%; *P*-value = 0.398).

### Treatment counselling

The proportion of caretakers to whom the HCWs mentioned both the number of doses a day and the number of days for all the relevant oral medicines prescribed for treating the child at home was 77% (473/612) at baseline, 92% (1046/1143) in the control districts and 88% (506/576) in the intervention districts with no evidence for a difference between trial arms (cluster-level mean difference = − 4.1%; *P*-value = 0.355) (Table 4). For all oral medicines, both the number of doses per day and the number of days were mentioned by the HCWs to a high proportion of caretakers at baseline and in both trial arms (Table 12).

**Table 12:** Correct treatment counselling: Proportion of caretakers to whom the HCWs mentioned both the number of doses a day and the number of days for home-based treatment

Treatment	Baseline				Control arm				Intervention arm			
	N^a^	%	95%CI		N^a^	%	95%CI		N^a^	%	95%CI	
Oral antibiotics for pneumonia ^b^	290	**81.7**	70.3	89.4	538	**93.9**	90.9	95.9	228	**91.7**	88.8	93.8
Artemisinin-based Combinnation Therapy (ACT) ^c^	470	**93.6**	89.1	96.3	721	**96.4**	94.1	97.8	360	**96.7**	94.5	98.0
ORS	99	**46.5**	25.1	69.2	264	**86.7**	77.4	92.6	148	**84.5**	59.6	95.2
Zinc	99	**83.8**	74.9	90.0	268	**94.0**	91.7	95.7	153	**87.6**	63.9	96.6
Oral anti-infectious for dysentery ^d^	19	**57.9**	26.5	84.0	54	**83.3**	69.5	91.7	18	**94.4**	70.0	99.2
Deworming treatments ^e^	73	**80.8**	59.4	92.4	111	**94.6**	77.2	98.9	68	**89.7**	73.6	96.5
Iron/ folic acid	42	**47.6**	19.5	77.3	40	**92.5**	69.0	98.6	72	**83.3**	58.8	94.6
Plumpy nut or equivalent	28	**32.1**	17.6	51.3	16	**75.0**	41.0	92.8	27	**63.0**	55.8	69.6

^a^Number of children who were prescribed, by the HCWs, a given treatment (regardless of the classification)

^b^ Amoxicillin, Cotrimoxazole; ^c^ Artesunate + Amodiaquine, Artemether/ Lumefantrine; ^d^ Ciprofloxacin, Metronidazole; ^e^ Albendazole, Mebendazole

### Availability of essential oral medicines and equipment

The average proportion of essential oral medicines that were observed to be available at the health facilities was high: 98% at baseline, 94% in the control districts and 89% in the intervention districts (Table 13). However, deworming treatments, amoxicillin, ORS and multivitamins were less frequently available in the intervention districts compared to the control districts.

**Table 13:** Availability of essential oral medicines and equipment

	Baseline (*N* = 158)		Control arm (*N* = 292)		Intervention arm (*N* = 168)	
	N	%	N	%	N	%
**Oral medicine (availability observed with at least one unexpired)**						
Albendazole	158	**94.3**	290	**93.5**	166	**79.5**
Amoxicillin	158	**97.5**	291	**89.7**	166	**73.5**
Artesunate + Amodiaquine or Artemether/ Lumefantrine (ACT)	158	**99.4**	291	**99.3**	166	**97.6**
Ciprofloxacin	158	**99.4**	291	**97.3**	166	**94.6**
Cotrimoxazole	158	**99.4**	291	**99.3**	166	**98.2**
Iron and folic acid	157	**98.7**	291	**98.6**	165	**94.6**
Mebendazole	158	**98.1**	291	**97.6**	166	**89.2**
Metronidazole	158	**100.0**	290	**99.7**	166	**97.6**
Multivitamins	158	**95.6**	290	**75.9**	166	**68.1**
Oral Rehydration Salt (ORS)	158	**94.3**	291	**93.1**	166	**85.5**
Ready to use therapeutic food (RUTF)	158	**96.2**	289	**92.0**	166	**91.0**
Zinc	158	**95.6**	288	**93.1**	165	**87.3**
Vitamin A	158	**100.0**	290	**98.6**	164	**97.0**
Availability index of essential oral medicines (13 items)	158	**97.6**	291	**94.4**	166	**88.7**
**Equipment (availability observed or reported)**						
Source of electricity	158	**91.1**	292	**98.6**	168	**100.0**
Electricity without any power cut in the last 7 days	155	**41.9**	198	**33.3**	254	**66.7**
Baby weighing scale (graduation 100 g)	157	**100.0**	291	**99.3**	168	**100.0**
Children weighing scale (graduation 250 g)	157	**100.0**	291	**99.3**	168	**100.0**
Measuring rod	157	**100.0**	291	**99.3**	168	**100.0**
Mid-upper arm circumference tape	157	**100.0**	290	**99.7**	168	**100.0**
Thermometer	157	**100.0**	256	**99.6**	166	**99.4**
Rapid Diagnostic Test	157	**89.8**	291	**94.9**	167	**98.2**
Source of clean water	158	**91.8**	292	**96.2**	163	**97.0**
Spoons, cups and jugs to mix and administer ORS	158	**21.5**	289	**32.9**	168	**85.1**
Kit for intravenous injection	157	**94.3**	292	**64.7**	166	**31.3**
Single-use syringes with disposable needles	157	**100.0**	292	**99.0**	167	**99.4**
Refrigerator	152	**99.3**	287	**99.3**	168	**99.4**
Availability index of essential equipment (13 items)	158	**86.9**	292	**87.1**	161	**90.6**

With respect to essential equipment, availability at the health facilities was high: 87% at baseline, 87% in the control districts and 91% in the intervention districts. Better availability of electricity and equipment to administer ORS was observed in the intervention districts compared to the control districts.

### Explanatory analyses

#### Comparison of HCWs’ performance with and without use of IMCI paper-forms in the control districts

In order to assess whether the frequent use of IMCI paper-based form in the control districts had an effect on HCWs performance, primary and secondary outcomes in the control districts were compared between HCWs who were observed to use an IMCI paper-form and those who did not.

Surprisingly, HCWs who did not use an IMCI paper-form in the control districts seem to have better assessed danger signs than those who used a form: on average they performed 45% versus 22% of the recommended tasks respectively (Additional file 5). For all other outcomes, HCWs’ performance was similar between the two groups.

#### Agreement between HCWs and validation nurses’ clinical assessment

The square root of the mean square errors (RMSE) for the differences in child’s weight, height and temperature measurements between HCWs and validation nurses indicate differences of a small magnitude (< 1 kg, < 3 cm or < 1 °C) at baseline and in the trial arms (Additional file 6a). Higher RMSE were observed between HCWs and validation nurses’ measurements of mid-upper arm circumference (MUAC) (around 5 mm) and respiratory count (around 9 counts). All differences were fairly balanced between trial arms.

With respect to RDT results and caretakers’ answers about children’s key symptoms, actual agreement between HCWs and validation nurses were high (> 90%) at baseline and in the trial arms (Additional file 6b). The Kappa coefficients indicate that 90% or more of RDT results were in agreement beyond that expected by chance. The Kappa coefficients for caretakers’ answers range from 0.60 to 0.88.

### Secondary analyses

Excluding “contaminated” control districts for the period when they were contaminated removed a total of 173 consultations from the analysis and made little or no difference to the results (Additional file 7).

## Discussion

The IeDA intervention improved substantially HCW’s adherence to IMCI’s clinical assessment tasks (30% point increase on average across the intervention districts compared to the control districts), including the assessment of danger signs, which led to some overall increase in the proportion of children being correctly classified (around 10% point increase on average across the intervention districts compared to the control districts) but to no improvement in overall proportion of children receiving correct prescriptions. The intervention, however, appeared to have reduced over-prescription of antibiotics by 6 to 13% points.

Achieving correct classification depends, at least in part, on the clinical skills of the HCWs, which may be more difficult to improve than task adherence itself and may have limited the effect of the intervention on correct classification. Recent more advanced clinical charts, also built on electronic tools, such as electronic point-of-care tests (ePOCT) integrating malaria RDT, haemoglobin, pulse oximetry in all febrile patients and other tests (e.g. glucometer, C-reactive protein) in subgroups of them, have led to major improvements in febrile disease classification and a considerable reduction of antibiotic prescription [27].

In addition, using the eIMCI in Burkina Faso, improvements in classifications and prescriptions tended to be observed for less common conditions, such as dysentery and malnutrition, for which HCWs in the control districts performed relatively poorly. The data were also consistent with an improvement in danger sign identification, correct referrals/hospitalisations and management of severe malaria or severe febrile illness, although small numbers of such children preclude firm conclusions. For other, more common, conditions (e.g. malaria or pneumonia), HCWs in the control districts performed relatively well in classifying and prescribing the correct medicines, thus limiting the scope to detect an overall impact.

There were some notable differences between findings at baseline and in the control arm with respect to prevalence of pneumonia (27 and 16% respectively), malaria (69 and 55% respectively) and anaemia (13 and 7% respectively). At baseline and in the control arm, 33 and 18% of observations respectively occurred from January to March, during the peak of the pneumonia season. Observations during the malaria season (July to November) were less frequent at baseline (49%) compared to the control arm (61%). However, the higher prevalence at baseline is consistent with the higher proportion of positive RDT: 82% of RDTs were positive at baseline compared to 66% during the control steps. These results may reflect a more intense malaria season during the baseline steps. This could also explain the difference in anaemia prevalence, which is associated with malaria.

Our findings are broadly consistent with the limited evidence available on the effectiveness of eCDSS for improving adherence to IMCI (eIMCI). In 18 primary facilities in four districts of Tanzania, only 21% of children had all ten critical IMCI tasks assessed under paper-based IMCI compared to 71% under eIMCI (*p* < 0.001) [14]. In two basic health centres in the Kabul province of Afghanistan, only 24% of children underwent a physical examination in line with IMCI at baseline compared to 84% after 1 year of implementation (*p* < 0.05) [17]. Comparison of HCWs classifications with classifications given by an independent nurse in Tanzania showed that the electronic protocol improved overall correct classification: 83% under paper-based IMCI compared to 91% under eIMCI (p < 0.001) [14]. In Afghanistan, only 35% of children received a treatment in line with HCWs’ classifications at baseline compared to 99% after 1 year of implementation [17]. Reduction in over-prescriptions of antibiotic have also been reported using eIMCI in Afghanistan [17] and Tanzania [15].

In Burkina Faso, interviews with HCWs indicated that IeDA was well accepted, in particular with respect to the usefulness of the eCDSS in guiding through the clinical assessment (Blanchet K et al.: Realist evaluation of the Integrated electronic Diagnostic Approach (IeDA) for the management of childhood illness at primary health facilities in Burkina Faso, submitted). In Ghana, South Africa and Tanzania, HCWs reported similar opinions [13, 16]. Nevertheless, our realistic evaluation in Burkina Faso also revealed contextual factors that may have limited the effect of the IeDA intervention. First, staff turnover was reported to be common by district managers, in particular in remote rural facilities where most HCWs do not want to spend more than a few years. A visit in July 2017 in all intervention facilities revealed that around a third of HCWs (36%) had been changed within the last 12 months and that a relatively large proportion (36%) of HCWs had not benefited from the eIMCI training (Blanchet K et al.: Realist evaluation of the Integrated electronic Diagnostic Approach (IeDA) for the management of childhood illness at primary health facilities in Burkina Faso, submitted). Second, while supervision and audit with feedback can be effective in improving performance [28–30], monthly supervision visits planned under the IeDA intervention in Burkina Faso faced challenges. The district management teams reported limited budget, access to vehicles and time to dedicate to these visits (Blanchet K et al.: Realist evaluation of the Integrated electronic Diagnostic Approach (IeDA) for the management of childhood illness at primary health facilities in Burkina Faso, submitted).

In addition to incomplete coverage of the IeDA intervention, while pressure from children’s caretakers, sometimes reported during interviews with HCWs (Blanchet K et al.: Realist evaluation of the Integrated electronic Diagnostic Approach (IeDA) for the management of childhood illness at primary health facilities in Burkina Faso, submitted), may have limited the reduction in over-prescription of antibiotics, the relatively lower availability of some essential medicines, such as amoxicillin and ORS, in the intervention facilities compared to the control facilities may have limited improvement in correct prescriptions for pneumonia, severe acute malnutrition without complications and diarrhoea. Multiple conditions may also have influenced the medicines prescribed. Across baseline and trial arms, about a third or more of children were diagnosed with two or more classifications. In Tanzania, a large know-do gap was observed, and a lack of knowledge was not the only constraint identified for improved performance. HCWs’ weak belief in the importance of following guidelines and confidence in their own experience, lack of intrinsic motivation, and physical or cognitive “overload” were also reported, with poor remuneration contributing to several of these factors [31].

### Limitations

Some limitations of our evaluation should be acknowledged. First, the “gold standard” classifications were provided by a repeat consultation after the initial consultation and it is possible that the clinical status of some children (e.g. respiratory rate, temperature, current convulsions) may have changed in the interval between the two. Therefore, we should not expect full agreement between HCWs and validation nurses. Our “gold standard” is certainly less than perfect, and this would tend to reduce the apparent magnitude of any improvement in classifications.

Second, it is likely that the behaviour of HCWs was impacted by the fact that they were observed [32]. The high proportion of HCWs observed using IMCI paper-forms in the control districts (68% overall) compared to routine practice (less than 8% of under-five consultations in 2012 [33]) suggests that HCWs in this arm were motivated to perform better than usual. Even if HCWs in the control districts who used IMCI paper-forms did not seem to have performed better compared to those who did not use IMCI paper-forms, repeated observations might explain improvements in some indicators from baseline to control steps, for instance adherence to assessment of danger signs (18% at baseline compared to 34% during control steps). Nevertheless, the behaviour of HCWs in the intervention districts may also have been affected by the presence of observers. Therefore, our findings may over-estimate how well HCWs perform in the absence of an observer, but it is difficult to assert whether or in which direction this may have affected the comparison of intervention and control districts.

Third, the initial evaluation design was not followed. In particular, rolling out the intervention to all districts as planned would have led to more data in the intervention arm, which could have strengthen our findings. In addition, the evaluation design could not address the multi-faceted nature of the intervention and evolving version of the eCDSS. It is therefore not possible to distinguish which component of the intervention led to observed improvements or whether improvements were the result of the combination of components.

Lastly, with respect to statistical analyses, multiple comparisons between arms were performed and can increase the overall error in hypothesis testing, so that *P*-values should be interpreted with caution. The small number of clusters per trial arm precluded using random effects models on individual level data, thus limiting our ability to control for individual child-level factors.

## Conclusion

To conclude, the IeDA intervention was well accepted and improved substantially HCW’s adherence to IMCI clinical assessment which led to some improvements in overall correct classifications but little or no improvement in overall correct prescriptions. Nevertheless, substantial improvements were observed in correct classifications and prescriptions for dysentery and malnutrition. To some degree, we also observed an improvement in danger sign identification, correct referrals/hospitalisations and management of severe malaria, although small numbers prevent firm conclusions. For the most common conditions, HCWs in the control districts, who may have been influenced by a Hawthorne effect, performed relatively well, limiting the scope to detect an overall impact.

HCWs’ practices are complex behaviours that have many potential contextual and intrinsic influences. Lower availability of some essential medicines in the intervention districts was observed and our realistic evaluation concurrently reported staff turnover and incomplete coverage of training and supervision which may have limited the effect of the IeDA intervention on correct classification and prescription. Task adherence may be easier to achieve than correct classifications which require clinical skills. In the context of national scaling up, disparities between regions exist in terms of structures, staff and resources. Nevertheless, complete coverage of the eIMCI training could be achieved by its integration into the initial nursing curriculum. Supervision will inevitably require resources but also management capacity to deal with relationships, organisation culture and HCWs’ professional norms, experiences and motivation (Blanchet K et al.: Realist evaluation of the Integrated electronic Diagnostic Approach (IeDA) for the management of childhood illness at primary health facilities in Burkina Faso, submitted).

## Supplementary Information

**Additional file 1.** Definition of primary and secondary outcomes.

**Additional file 2.** Definition of other reported outcomes.

**Additional file 3.** Completed CONSORT checklist.

**Additional file 4.** Actual roll-out of the IeDA intervention with step dates and number (N) of children aged 2–59 months observed at each step in each district.

**Additional file 5.** Comparison of HCWs’ performance with and without use of IMCI paper-forms in the control arm. (DOCX 18 kb)

**Additional file 6.** Agreement between HCWs and validation nurses’ children measurements. 6b: Agreement between HCWs and validation nurses’ RDT results and caretakers’ answers.

**Additional file 7.** Primary and secondary outcomes (secondary analyses, excluding “contaminated” control districts).

## Data Availability

The datasets used and/or analysed during the current study are available from the corresponding author on reasonable request.

## Abbreviations

ACT: Artemisinin-based Combination Therapy
eCDSS: electronic Clinical Decision Support System
ePOCT: electronic point-of-care tests
HCW: Health Care Worker
IeDA: integrated eDiagnosis Approach
IMCI: Integrated Management of Childhood illness
LMIC: Low- and Middle-Income Countries
LSHTM: London School of Hygiene and Tropical Medicine
MoH: Ministry of Health
MUAC: Mid-Upper Arm Circumference
PBF: performance-Based Financing
RDT: Rapid Diagnostic Test
RMSE: square root of the mean square errors
Tdh: Terre des hommes foundation
