# Supplementary material for: An Integrated eDiagnosis Approach (IeDA) versus standard IMCI for assessing and managing childhood illness in Burkina Faso: a stepped-wedge cluster randomised trial
Source: BMC Health Serv Res. 2021 Apr 16;21:354. doi: 10.1186/s12913-021-06317-3 (PMC8052659; doi:10.1186/s12913-021-06317-3)
Supplement: Supplementary file 1 — Additional file 1. Definition of primary and secondary outcomes. [file 12913_2021_6317_MOESM1_ESM.docx]

**Additional file 1: Definition of primary and secondary outcomes**

***Overall adherence to clinical assessment tasks:*** An index was calculated as the average proportion of required tasks (either questions to address to the child’s caretaker or clinical examinations) that were observed to be completed across IMCI charts. For each consultation, the proportion of completed tasks was computed and the arithmetic mean was then computed across all consultations to give the overall adherence index. While some tasks are required for all children, some are conditional tasks (e.g. ask about duration of a cough if the caretaker reported a cough). Conditional tasks were included in the calculation of the index only if the condition was met.

***Adherence to assessment of danger signs:*** An index was calculated as above but restricted to the chart of interest.

***Correct identification of at least one danger sign*** was defined as the proportion of children recorded, by the validation nurses, as having at least one danger sign who were also identified, by the HCWs, with at least one danger sign. Although there was no obvious clinical examination to observe HCWs’ assessment of current convulsions or lethargy/unconsciousness (making these tasks excluded from the adherence index), the independent nurses who observed consultations recorded whether the child convulsed during the consultation or was lethargic/unconscious. Thus, unlike the computation of the adherence index, the correct identification of at least one danger sign was computed accounting for all four danger signs.

***Overall correct classification*** was defined as the proportion of children recorded, by the validation nurse, with x given classifications who were also classified, by the HCW, with the same x given classifications.

Overall correct classification was computed both ***ignoring the severity of the classifications*** (upon the advice of the trial’s scientific advisory committee), e.g. combining severe or not severe pneumonia, and ***accounting for the severity of the classifications***.

***Overall correct prescription*** was defined as the proportion of children with x given classifications who were prescribed at least all the recommended medicines (i.e. over-prescription was not penalised and data on dosage were not collected) (table below). In 2015, the national IMCI guidelines changed recommended prescriptions for some conditions (e.g. to treat pneumonia, cotrimoxazole or amoxicillin were recommended from 2012 to 2014, amoxicillin only from 2015), and correct prescriptions for these conditions were defined to allow both recommended prescriptions to be correct (e.g. to treat pneumonia, cotrimoxazole or amoxicillin).

Overall correct prescription was computed both ***according to the HCWs and validation nurses’ classifications***.

***Overall correct referral or hospitalisation*** was defined as the proportion of children in need of referral to a higher-level facility or hospitalisation who were actually referred or hospitalised. Children were considered in need of referral or hospitalisation if they were identified with at least one danger sign or classified with a severe classification warranting referral or hospitalisation (table below). Due to the poor road network and usually very long distance to the closest hospital in Burkina Faso, hospitalisation at the primary facility is common and was considered as a practice in line with the IMCI guidelines.

Overall correct referral or hospitalisation was computed both ***according to the HCWs and validation nurses’ assessment***.

***Overall correct treatment counselling*** was defined as the proportion of caretakers to whom the HCW mentioned both the number of doses a day and the number of days of treatment among all children who were prescribed, by the HCWs, an oral medicine for treating the child at home.

**Correct prescriptions and referral or hospitalisation**

| Classification | 2012 recommendation | 2015 recommendation | Definition of correct prescription | In need of referral/ hospitalisation |
| --- | --- | --- | --- | --- |
| Severe pneumonia or very severe disease | Ampicillin IM/IV & Gentamicin IM/IV | | Ampicillin IM/IV & Gentamicin IM/IV | Yes |
| Pneumonia | 1^st^ line: Cotrimoxazole, 2^nd^ line: Amoxicillin | Amoxicillin | Cotrimoxazole or Amoxicillin | No |
| Severe dehydration with other severe classification | Plan C: Ringer lactate IV or ORS (per naso-gastric tube or per os) | | Ringer lactate IV or ORS (per naso-gastric tube or per os) | Yes |
| Severe dehydration without other severe classification | Plan C: Ringer lactate IV or ORS (per naso-gastric tube or per os) | | Ringer lactate IV or ORS (per naso-gastric tube or per os) | No |
| Dehydration with other severe classification | Ringer lactate IV or ORS (per naso-gastric tube or per os) | | Ringer lactate IV or ORS (per naso-gastric tube or per os) | Yes |
| Dehydration without other severe classification | Plan B: ORS per os & zinc | | ORS per os & zinc | No |
| Diarrhoea with no dehydration | Plan A: (ORS per os or increase liquids) & zinc | | Zinc | No |
| Severe persistent diarrhoea | Ringer lactate IV or ORS (per naso-gastric tube or per os) | | Ringer lactate IV or ORS (per naso-gastric tube or per os) | Yes |
| Persistent diarrhoea | Multivitamins & zinc | | Multivitamins & zinc | No |
| Dysentery | (Ciprofloxacin or (Ciprofloxacin & Metronidazole)) & zinc | | Ciprofloxacin & zinc | No |
| Severe malaria or severe febrile illness | Quinine IM/IV & Ampicillin IM/IV & Gentamicin IM/IV | (1^st^ line: Artesunate IM/IV or Artemether IM/IV, 2^nd^ line: Quinine IM/IV) & (Ampicillin IM/IV & Gentamicin IM/IV) | (Artesunate IM/IV or Artemether IM/IV or Quinine IM/IV) & (Ampicillin IM/IV & Gentamicin IM/IV) | Yes |
| Malaria | Artesunate + Amodiaquine or Artemether/ Lumefantrine | | Artesunate + Amodiaquine or Artemether/ Lumefantrine | No |
| Severe acute malnutrition with complications (any danger sign or other severe classification) | Ampicillin IM/IV | | Ampicillin IM/IV | Yes |
| Severe acute malnutrition without complications | Amoxicillin & (Mebendazole or Albendazole if age>11 months & no dose in the past 6 months) & RUTF | | Amoxicillin & RUTF ∔ | No |
| Moderate acute malnutrition | (Vitamin A if age > 5 & no dose in the past 6 months) & Iron/Folic acid & (Mebendazole or Albendazole if age>11 months & no dose in the past 6 months) & RUTF | (Vitamin A if age > 5 & no dose in the past 6 months) & Iron/Folic acid & (Mebendazole or Albendazole if age>11 months & no dose in the past 6 months) & RUSF | Iron/Folic acid & RUSF/RUTF ∔ | No |
| ∔ Vitamin A, Albendazole or Mebendazole not accounted for in the analysis due to lack of information about uptake in the past 6 months | | |  |  |
| ORS: Oral Rehydration Salt, RUSF: Ready to Use Supplementary Food, RUTF: Ready to Use Therapeutic Food | | |  |  |
