# Supplementary material for: An Integrated eDiagnosis Approach (IeDA) versus standard IMCI for assessing and managing childhood illness in Burkina Faso: a stepped-wedge cluster randomised trial
Source: BMC Health Serv Res. 2021 Apr 16;21:354. doi: 10.1186/s12913-021-06317-3 (PMC8052659; doi:10.1186/s12913-021-06317-3)
Supplement: Supplementary file 2 — Additional file 2. Definition of other reported outcomes. [file 12913_2021_6317_MOESM2_ESM.docx]

**Additional file 2: Definition of other reported outcomes**

***Sensitivity of the HCW’s classification*** was defined as the proportion of children recorded by the validation nurse with a given classification who were also classified by the HCW with the same classification (“true positive rate”).

***Specificity of the HCW’s classification*** was defined as the proportion of children not recorded by the validation nurse with a given classification who were also not classified, by the HCW, with the same classification (“true negative rate”).

***Over-prescription of antibiotics and antimalarials*** was defined as the proportion of children who were not in need of an antibiotic and an antimalarial according to their classification but who were actually prescribed it (table below). Over-prescription was computed both according to the HCWs and validation nurses’ classifications.

***Overall availability index of essential oral medicines and equipment*** were defined as the average proportion of essential items that were observed to be available at the health facility. At each health facility visit, the proportions of available essential items were computed, and the arithmetic means were then computed across all visits to give the overall availability index.

**Over-prescriptions**

| Medicine | Definition of over-prescription |
| --- | --- |
| Amoxicillin | Prescribed in the absence of pneumonia, severe acute malnutrition without complications, measles with eye or mouth complications, measles, acute ear infection, chronic ear infection |
| Ampicillin (injectable) | Prescribed in the absence of severe pneumonia, severe malaria, severe acute malnutrition with complications, severe and complicated measles and mastoiditis |
| Artemisinin-based combination therapy | Prescribed in the absence of malaria and anaemia |
| Artesunate or Artemether (injectable) | Prescribed in the absence of severe malaria and severe anaemia |
| Ciprofloxacin | Prescribed in the absence of dysentery |
| Cotrimoxazole | Prescribed in the absence of pneumonia, confirmed and symptomatic HIV infection, confirmed HIV infection, probable and symptomatic HIV infection, possible HIV infection/ exposure to HIV |
| Gentamycin (injectable) | Prescribed in the absence of severe pneumonia, severe malaria, severe and complicated measles and mastoiditis |
| Metronidazole | Prescribed in the absence of dysentery |
| Quinine (injectable) | Prescribed in the absence of severe malaria and severe anaemia |
