# Supplementary material for: An Integrated eDiagnosis Approach (IeDA) versus standard IMCI for assessing and managing childhood illness in Burkina Faso: a stepped-wedge cluster randomised trial
Source: BMC Health Serv Res. 2021 Apr 16;21:354. doi: 10.1186/s12913-021-06317-3 (PMC8052659; doi:10.1186/s12913-021-06317-3)
Supplement: Supplementary file 4 — Additional file 4. Actual roll-out of the IeDA intervention with step dates and number (N) of children aged 2–59 months observed at each step in each district. [file 12913_2021_6317_MOESM4_ESM.docx]

**Additional file 4: Actual roll-out of the IeDA intervention with step dates and number (N) of children aged 2-59 months observed at each step in each district**

| District | IeDA full implementation completion dates | Step & Data collection dates | | | | | | | | Total by district |
| --- | --- | --- | --- | --- | --- | --- | --- | --- | --- | --- |
|  |  | 1 | 2 | 3 | 4 | 5 | 6 | 7 | 8 |  |
|  |  | 22 Sep 2014 - 29 Jan 2015 | 26 Feb 2015 - 29 Apr 2015 | 07 Jul 2015 - 30 Nov 2015 | 20 Dec 2015 - 08 Apr 2016 | 14 Jun 2016 - 16 Oct 2016 | 16 Jan 2017 - 13 Apr 2017 | 14 Apr 2017 - 10 Aug 2017 | 11 Sep 2017 - 11 Nov 2017 |  |
| Gourcy | Dec 2017 | N = 75 | N = 26 | N = 37 | N = 52 | N = 32 | N = 1 | N = 40 | N = 55 | N = 318 |
| Dedougou | - | N = 95 | N = 41 | N = 60 | N = 52 | N = 65 | N = 52 | N = 35 | N = 73 | N = 473 |
| Boromo | - | N = 86 | N = 36 | N = 59 | N = 17 | N = 59 | N = 47 | N = 35 | N = 44 | N = 383 |
| Nouna | - | N = 49 | N = 23 | N = 51 | N = 27 | N = 67 | N = 41 | N = 31 | N = 57 | N = 346 |
| Ouahigouya | Aug 2016 | N = 78 | N = 26 | N = 57 | N = 17 | N = 46 | N = 23 | N = 21 | N = 19 | N = 287 |
| Titao | Apr 2016 | N = 47 | N = 22 | N = 57 | N = 30 | N = 46 | N = 40 | N = 2 | N = 55 | N = 299 |
| Solenzo | Dec 2015 | N = 23 | N = 17 | N = 47 | N = 42 | N = 82 | N = 50 | N = 37 | N = 61 | N = 359 |
| Toma | Jul 2015 | N = 17 | N = 25 | N = 38 | N = 20 | N = 52 | N = 37 | N = 27 | N = 43 | N = 259 |
| Total by step | | N = 470 | N = 216 | N = 406 | N = 257 | N = 449 | N = 291 | N = 228 | N = 407 | N = 2,724 |
| Districts shaded in dark green had full implementation of the IeDA intervention | | | | |  |  |  |  |  |  |
| Districts shaded in light green had partial implementation of the IeDA intervention ("contaminated" control districts) | | | | | | |  |  |  |  |
