# Supplementary material for: An Integrated eDiagnosis Approach (IeDA) versus standard IMCI for assessing and managing childhood illness in Burkina Faso: a stepped-wedge cluster randomised trial
Source: BMC Health Serv Res. 2021 Apr 16;21:354. doi: 10.1186/s12913-021-06317-3 (PMC8052659; doi:10.1186/s12913-021-06317-3)
Supplement: Supplementary file 5 — Additional file 5. Comparison of HCWs’ performance with and without use of IMCI paper-forms in the control arm. (DOCX 18 kb) [file 12913_2021_6317_MOESM5_ESM.docx]

**Additional file 5: Comparison of HCWs’ performance with and without use of IMCI paper-forms in the control arm**

| **Adherence to clinical assessment** | Without IMCI paper-form | | | | With IMCI paper-form | | | |
| --- | --- | --- | --- | --- | --- | --- | --- | --- |
|  | N | % | 95%CI | | N | % | 95%CI | |
| Overall adherence (33 tasks) | 307 | **57.1** | 53.5 | 60.7 | 916 | **50.2** | 44.8 | 55.6 |
| Adherence to danger signs' assessment (3 tasks) | 307 | **45.1** | 33.1 | 57.1 | 916 | **22.2** | 11.6 | 32.8 |
|  |  |  |  |  |  |  |  |  |
| **Identification of at least one danger sign** (proportion of children correctly identified with at least one danger sign) | Without IMCI paper-form | | | | With IMCI paper-form | | | |
|  | N^☨^ | % | 95%CI | | N^☨^ | % | 95%CI | |
|  | 6 | **66.7** | 45.0 | 83.0 | 18 | **50.0** | 22.2 | 77.8 |
| ☨Number of children identified, by the validation nurses, with a given danger sign | | | |  |  |  |  |  |
|  |  |  |  |  |  |  |  |  |
| **Overall correct classification** (proportion of children correctly classified with x given classifications) | Without IMCI paper-form | | | | With IMCI paper-form | | | |
|  | N^☨^ | % | 95%CI | | N^☨^ | % | 95%CI | |
| Accounting for the severity of classifications | 240 | **71.7** | 60.0 | 81.0 | 703 | **67.4** | 63.4 | 71.2 |
| Ignoring the severity of the classifications | 240 | **74.6** | 64.4 | 82.7 | 703 | **71.0** | 65.8 | 75.7 |
| ☨ Number of children classified, by the validation nurses, with x given classification | | | |  |  |  |  |  |
|  |  |  |  |  |  |  |  |  |
| **Overall correct prescription** (proportion of children who received at least all the recommended prescriptions) | Without IMCI paper-form | | | | With IMCI paper-form | | | |
|  | N^☨^ | % | 95%CI | | N^☨^ | % | 95%CI | |
| According to the HCWs' classifications | 238 | **77.7** | 60.5 | 88.8 | 733 | **77.5** | 73.5 | 81.0 |
| According to the validation nurses' classifications | 240 | **67.5** | 51.7 | 80.1 | 703 | **63.9** | 60.3 | 67.3 |
| ☨Number of children classified, by the HCWs or by the validation nurses, with x given classification | | | | | |  |  |  |
|  |  |  |  |  |  |  |  |  |
| **Overall correct referral/hospitalisation** (proportion of children in need of referral/hospitalisation who were actually referred/hospitalised) | Without IMCI paper-form | | | | With IMCI paper-form | | | |
|  | N^☨^ | % | 95%CI | | N^☨^ | % | 95%CI | |
| According to the HCWs' classifications | 8 | **62.5** | 32.1 | 85.5 | 27 | **55.6** | 19.2 | 86.8 |
| According to the validation nurses' classifications | 6 | **50.0** | 23.3 | 76.7 | 25 | **52.0** | 33.9 | 69.6 |
| ☨Number of children identified, by the HCWs or the validation nurses, with at least one danger sign or a classification requiring referral/hospitalisation | | | | | | | | |
|  |  |  |  |  |  |  |  |  |
| **Overall correct treatment counselling** (proportion of caretakers who received information on home-based prescription) | Without IMCI paper-form | | | | With IMCI paper-form | | | |
|  | N^☨^ | % | 95%CI | | N^☨^ | % | 95%CI | |
|  | 255 | **87.5** | 81.0 | 91.9 | 779 | **92.8** | 91.0 | 94.3 |
| ☨ Number of children who were prescribed, by the HCWs, x given treatment (regardless of the classification) | | | | | | |  |  |
