# Supplementary material for: An Integrated eDiagnosis Approach (IeDA) versus standard IMCI for assessing and managing childhood illness in Burkina Faso: a stepped-wedge cluster randomised trial
Source: BMC Health Serv Res. 2021 Apr 16;21:354. doi: 10.1186/s12913-021-06317-3 (PMC8052659; doi:10.1186/s12913-021-06317-3)
Supplement: Supplementary file 6 — Additional file 6. Agreement between HCWs and validation nurses’ children measurements. 6b: Agreement between HCWs and validation nurses’ RDT results and caretakers’ answers. [file 12913_2021_6317_MOESM6_ESM.docx]

**Additional file 6a: Agreement between HCWs and validation nurses' children measurements**

|  | Baseline | | | Control arm | | | Intervention arm | | |
| --- | --- | --- | --- | --- | --- | --- | --- | --- | --- |
|  | N | mean (SD) | RMSE | N | mean (SD) | RMSE | N | mean (SD) | RMSE |
| Difference in weight (kg) | 676 | 0.02 (0.70) | 0.70 | 1,320 | 0.08 (0.75) | 0.76 | 687 | 0.01 (0.45) | 0.45 |
| Difference in height (cm) | 580 | 0.12 (2.43) | 2.43 | 1,213 | 0.07 (2.78) | 2.78 | 692 | 0.34 (2.81) | 2.83 |
| Difference in MUAC (mm) | 544 | -0.25 (5.62) | 5.62 | 1,087 | -0.77 (4.69) | 4.75 | 600 | -0.21 (3.87) | 3.87 |
| Difference in temperature (°C) | 669 | -0.03 (0.53) | 0.53 | 1,203 | -0.09 (0.52) | 0.53 | 646 | -0.06 (0.54) | 0.55 |
| Difference in respiratory count | 169 | 0.18 (9.51) | 9.48 | 226 | -0.47 (9.51) | 9.50 | 285 | 0.51 (8.66) | 8.66 |

**Additional file 6b: Agreement between HCWs and validation nurses' RDT results and caretakers' answers**

|  | Baseline | | | Control arm | | | Intervention arm | | | |
| --- | --- | --- | --- | --- | --- | --- | --- | --- | --- | --- |
|  | N | Actual agreement (%) | Kappa coefficient | N | Actual agreement (%) | Kappa coefficient | N | Actual agreement (%) | | Kappa coefficient |
| RDT result | 428 | 97.0 | 0.90 | 913 | 96.9 | 0.93 | 530 | 96.2 | | 0.92 |
| Caretaker's answer about cough/difficult breathing | 648 | 91.1 | 0.82 | 1,267 | 90.8 | 0.81 | 690 | 94.1 | | 0.88 |
| Caretaker's answer about diarrhoea | 647 | 90.4 | 0.75 | 1,237 | 90.9 | 0.77 | 686 | 94.6 | | 0.86 |
| Caretaker's answer about blood in stool | 76 | 97.4 | 0.82 | 162 | 91.4 | 0.60 | 150 | 97.3 | | 0.80 |
| Caretaker's answer about fever/history of fever | 575 | 91.3 | 0.66 | 1,286 | 91.8 | 0.71 | 691 | 93.3 | | 0.75 |
| Too few rating categories for answers about able to drink/breastfeed, vomit everything, recent convulsion, dark/not abundant urine, abnormal bleeding | | | | | | | | |  |  |
