# Supplementary material for: An Integrated eDiagnosis Approach (IeDA) versus standard IMCI for assessing and managing childhood illness in Burkina Faso: a stepped-wedge cluster randomised trial
Source: BMC Health Serv Res. 2021 Apr 16;21:354. doi: 10.1186/s12913-021-06317-3 (PMC8052659; doi:10.1186/s12913-021-06317-3)
Supplement: Supplementary file 7 — Additional file 7. Primary and secondary outcomes (secondary analyses, excluding “contaminated” control districts). [file 12913_2021_6317_MOESM7_ESM.docx]

**Additional file 7: Primary and secondary outcomes (secondary analyses, excluding "contaminated" control districts)**

| **Adherence to IMCI's clinical assessment** | Baseline | | | | Control arm | | | | Intervention arm | | | | Cluster-level mean difference between arms | P-value* |
| --- | --- | --- | --- | --- | --- | --- | --- | --- | --- | --- | --- | --- | --- | --- |
|  | N | % | 95%CI | | N | % | 95%CI | | N | % | 95%CI | |  |  |
| Overall adherence (13 to 33 tasks) | 661 | **48.0** | 44.3 | 51.8 | 1,195 | **51.8** | 47.2 | 56.5 | 695 | **79.3** | 72.7 | 85.9 | **32.4** | 0.002 |
| Adherence to danger signs' assessment (3 tasks) | 661 | **18.4** | 11.6 | 25.1 | 1,195 | **28.1** | 21.0 | 35.2 | 695 | **95.2** | 90.0 | 99.9 | **76.9** | 0.002 |
|  |  |  |  |  |  |  |  |  |  |  |  |  |  |  |
| **Identification of at least one danger sign** (proportion of children correctly identified with at least one danger sign) | Baseline | | | | Control arm | | | | Intervention arm | | | | Individual-level difference between arms | P-value** |
|  | N^☨^ | % | 95%CI | | N^☨^ | % | 95%CI | | N^☨^ | % | 95%CI | |  |  |
|  | 24 | **66.7** | 47.2 | 81.7 | 24 | **54.2** | 31.9 | 74.8 | 16 | **75.0** | 50.5 | 89.8 | **20.8** | 0.318 |
| ☨Number of children identified, by the validation nurses, with a given danger sign | | | |  |  |  |  |  |  |  |  |  |  |  |
|  |  |  |  |  |  |  |  |  |  |  |  |  |  |  |
| **Overall correct classification** (proportion of children correctly classified with x given classifications) | Baseline | | | | Control arm | | | | Intervention arm | | | | Cluster-level mean difference between arms | P-value* |
|  | N^☨^ | % | 95%CI | | N^☨^ | % | 95%CI | | N^☨^ | % | 95%CI | |  |  |
| Accounting for the severity of classifications | 589 | **71.1** | 64.2 | 77.2 | 920 | **68.6** | 64.2 | 72.7 | 572 | **74.7** | 66.9 | 81.1 | **9.7** | 0.023 |
| Ignoring the severity of the classifications | 589 | **75.4** | 68.1 | 81.5 | 920 | **72.0** | 66.8 | 76.6 | 572 | **78.7** | 72.9 | 83.5 | **10.6** | 0.002 |
| ☨ Number of children classified, by the validation nurses, with x given classification | | | |  |  |  |  |  |  |  |  |  |  |  |
|  |  |  |  |  |  |  |  |  |  |  |  |  |  |  |
| **Overall correct prescription** (proportion of children who received at least all the recommended prescriptions) | Baseline | | | | Control arm | | | | Intervention arm | | | | Cluster-level mean difference between arms | P-value* |
|  | N^☨^ | % | 95%CI | | N^☨^ | % | 95%CI | | N^☨^ | % | 95%CI | |  |  |
| According to the HCWs' classifications | 597 | **76.2** | 68.4 | 82.6 | 950 | **77.6** | 71.6 | 82.6 | 567 | **77.1** | 71.6 | 81.8 | **-1.2** | 0.753 |
| According to the validation nurses' classifications | 590 | **66.3** | 61.1 | 71.1 | 920 | **65.0** | 59.6 | 70.0 | 572 | **68.5** | 58.8 | 76.9 | **6.9** | 0.195 |
| ☨Number of children classified, by the HCWs or by the validation nurses, with x given classification | | | | | |  |  |  |  |  |  |  |  |  |
|  |  |  |  |  |  |  |  |  |  |  |  |  |  |  |
| **Overall correct referral/hospitalisation** (proportion of children in need of referral/hospitalisation who were actually referred/hospitalised) | Baseline | | | | Control arm | | | | Intervention arm | | | | Individual-level difference between arms | P-value** |
|  | N^☨^ | % | 95%CI | | N^☨^ | % | 95%CI | | N^☨^ | % | 95%CI | |  |  |
| According to the HCWs' classifications | 34 | **61.8** | 47.8 | 74.0 | 34 | **58.8** | 27.9 | 84.1 | 41 | **61.0** | 21.5 | 89.9 | **2.2** | 0.999 |
| According to the validation nurses' classifications | 28 | **57.1** | 35.7 | 76.2 | 31 | **51.6** | 38.1 | 64.9 | 22 | **68.2** | 47.8 | 83.4 | **16.6** | 0.268 |
| ☨Number of children identified, by the HCWs or the validation nurses, with at least one danger sign or a classification requiring referral/hospitalisation | | | | | | | | | | |  |  |  |  |
|  |  |  |  |  |  |  |  |  |  |  |  |  |  |  |
| **Overall correct treatment counselling** (proportion of caretakers who received information on home-based prescription) | Baseline | | | | Control arm | | | | Intervention arm | | | | Cluster-level mean difference between arms | P-value* |
|  | N^☨^ | % | 95%CI | | N^☨^ | % | 95%CI | | N^☨^ | % | 95%CI | |  |  |
|  | 589 | **78.6** | 69.7 | 85.4 | 1,013 | **91.5** | 88.7 | 93.6 | 576 | **87.9** | 77.9 | 93.7 | **-4.7** | 0.289 |
| ☨ Number of children who were prescribed, by the HCWs, x given treatment (regardless of the classification) | | | | | |  |  |  |  |  |  |  |  |  |
|  |  |  |  |  |  |  |  |  |  |  |  |  |  |  |
| * t test on cluster-level summaries & accounting for the stepped wedge design; ** Fisher's exact test on individual-level data & ignoring clustering | | | | | | | | | | | | | | |
